# Ciliary Motion in the Pulmonary Mucous Membrane of Amphibia

**Published:** 1848-08

**Authors:** Thomas Williams

**Affiliations:** Swansea


*Ciliary Motion in the Pulmonary Mucous Membrane of Amphibia by* Thomas Williams, M. D., of Swansea.—I have recently demonstrated, on repeated occasions, and in the most conclusive manner, the existence of ciliary motion over the whole of the internal superficies of the lungs, in the newt, frog, lizard, and snake. I find that in the batrachian, ophidian, and saurian reptiles, ciliary vibration prevails over every part of the true respiratory localities of the pulmonary membrane, as distinguished from that of the air-passages. In a subsequent communication, I shall endeavour to give a more extended account of my observations.—*Lancet.*

